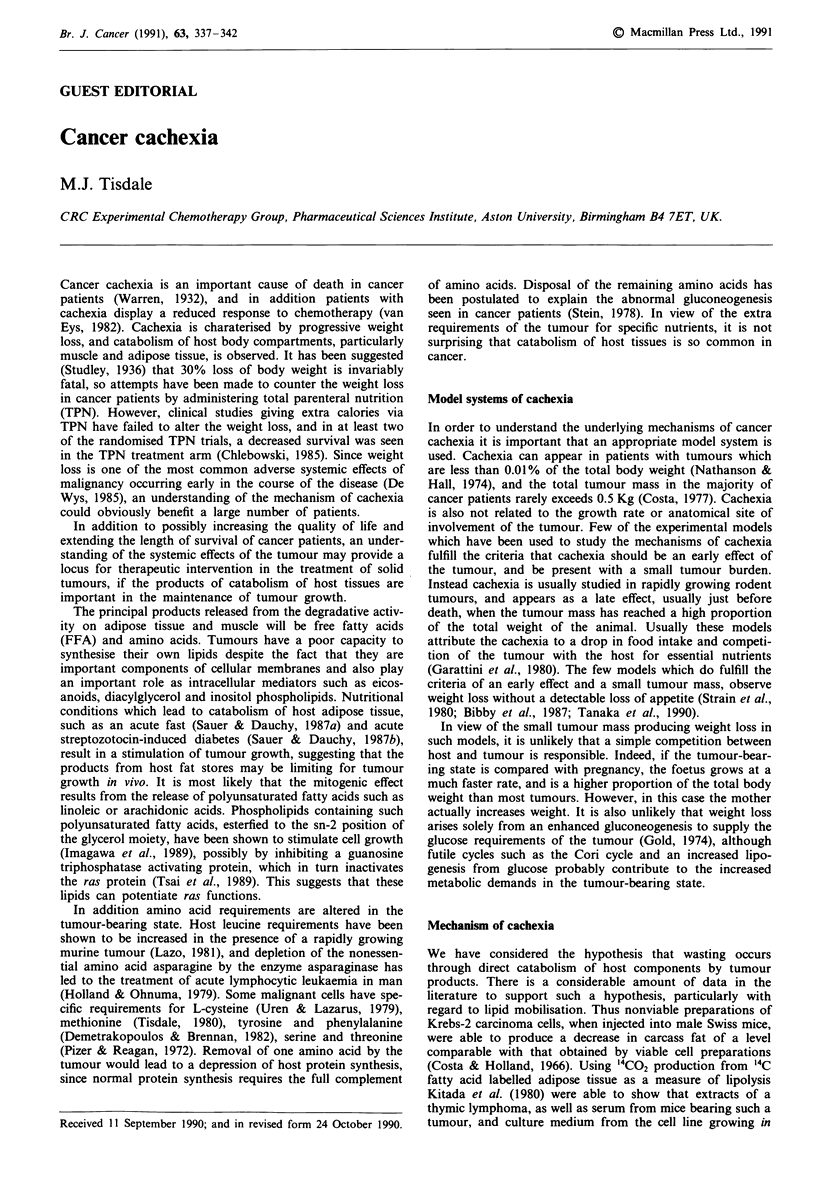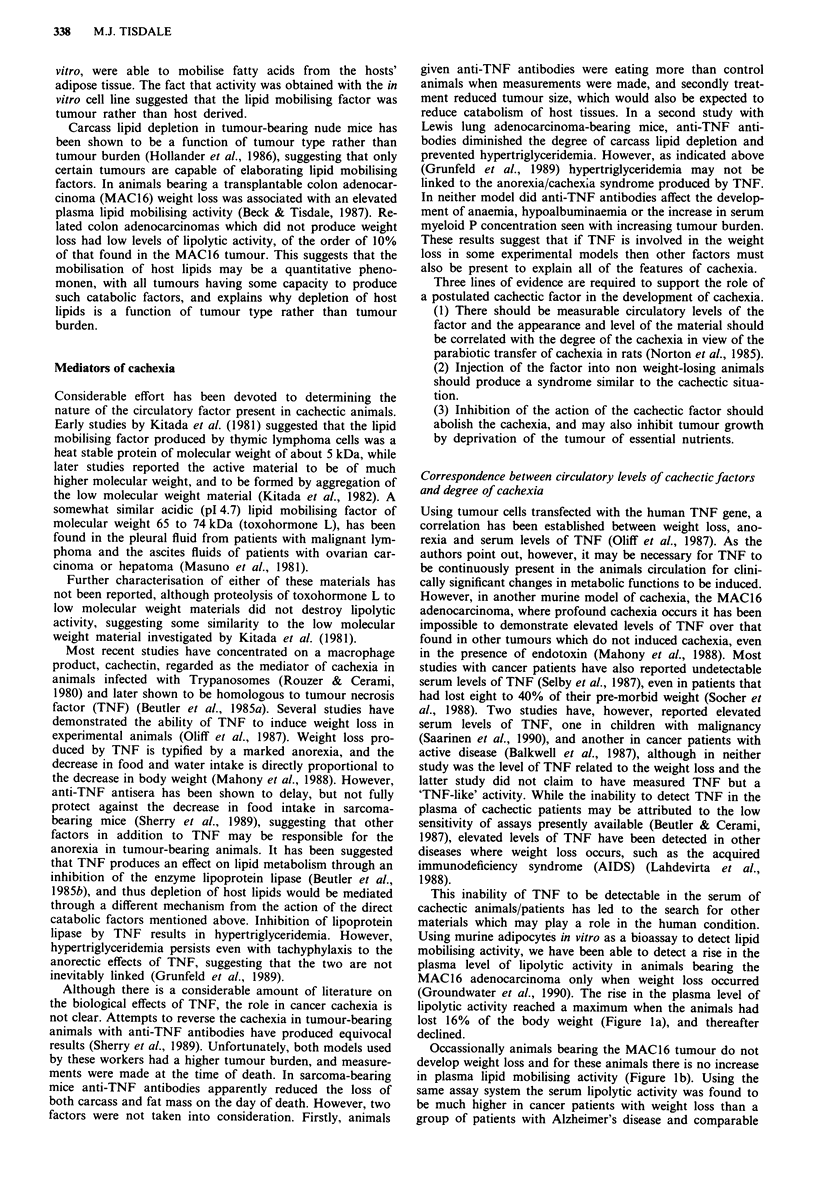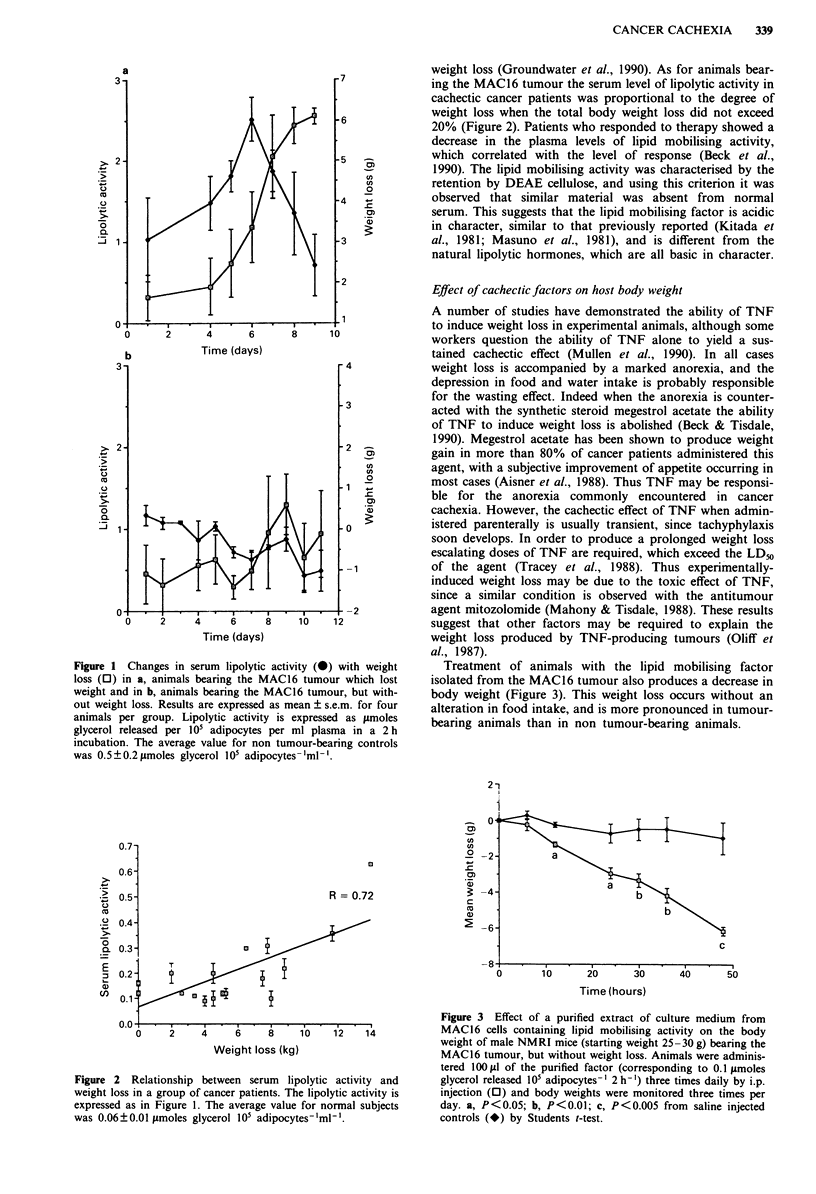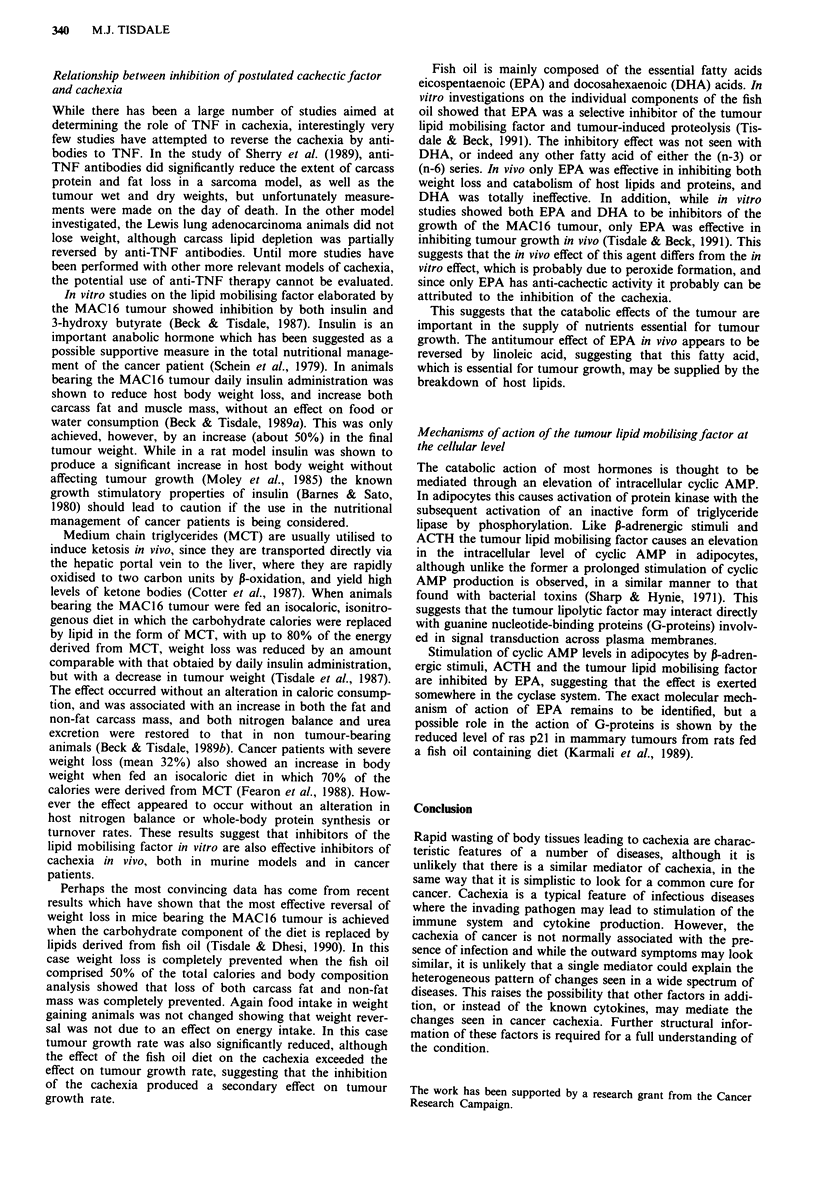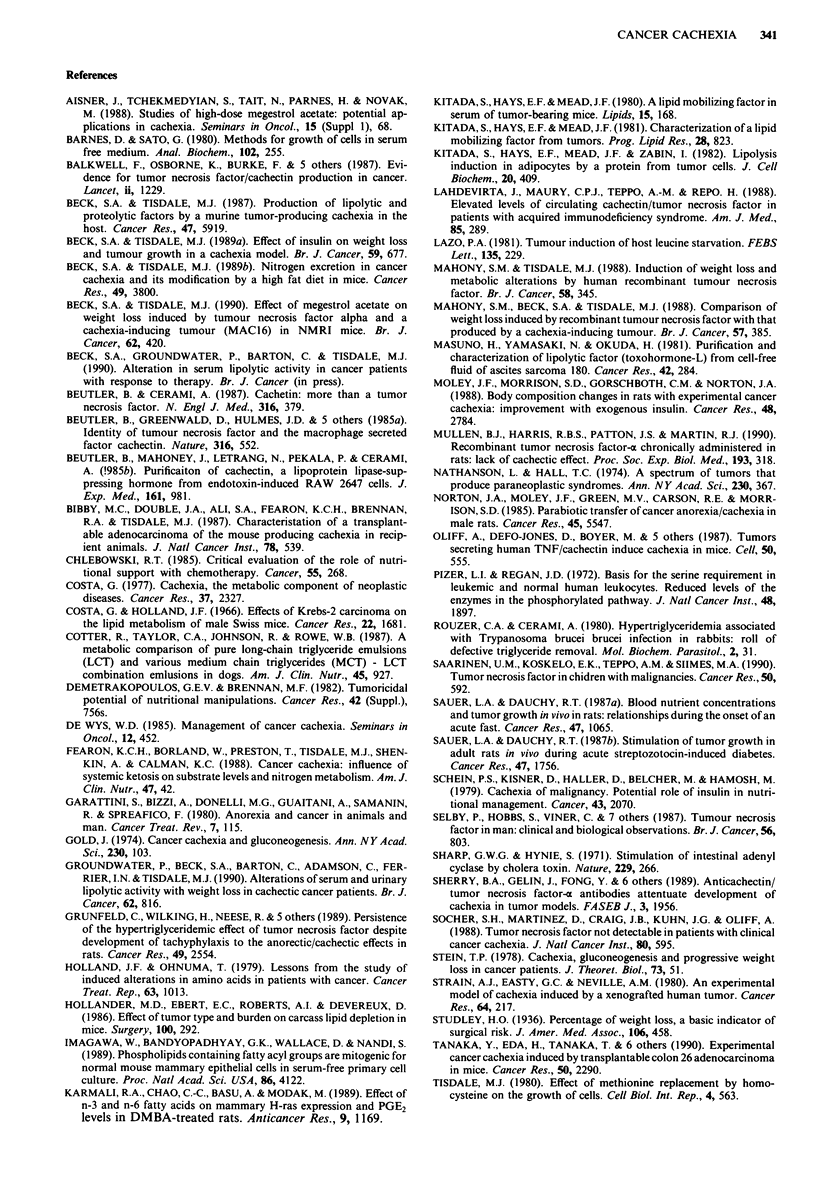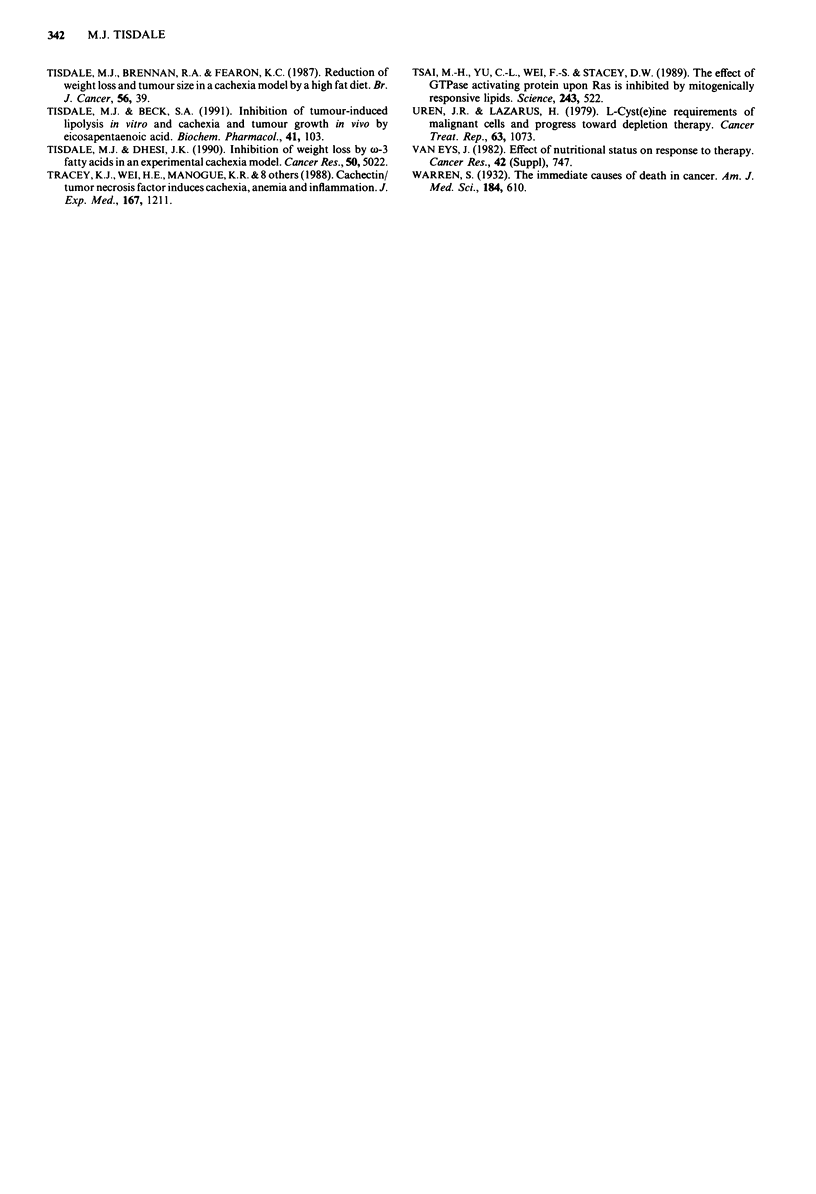# Cancer cachexia.

**DOI:** 10.1038/bjc.1991.80

**Published:** 1991-03

**Authors:** M. J. Tisdale


Br. J. Cancer (1991), 63, 337-342 © Macmillan Press Ltd., 1991

GUEST EDITORIAL

### Cancer cachexia

M.J. Tisdale

*CRC Experimental Chemotherapy Group, Pharmaceutical Sciences Institute, Aston University, Birmingham B4 7ET, UK.*

Cancer cachexia is an important cause of death in cancer patients (Warren, 1932), and in addition patients with cachexia display a reduced response to chemotherapy (van Eys, 1982). Cachexia is charaterised by progressive weight loss, and catabolism of host body compartments, particularly muscle and adipose tissue, is observed. It has been suggested (Studley, 1936) that 30% loss of body weight is invariably fatal, so attempts have been made to counter the weight loss in cancer patients by administering total parenteral nutrition (TPN). However, clinical studies giving extra calories via TPN have failed to alter the weight loss, and in at least two of the randomised TPN trials, a decreased survival was seen in the TPN treatment arm (Chlebowski, 1985). Since weight loss is one of the most common adverse systemic effects of malignancy occurring early in the course of the disease (De Wys, 1985), an understanding of the mechanism of cachexia could obviously benefit a large number of patients.

In addition to possibly increasing the quality of life and extending the length of survival of cancer patients, an understanding of the systemic effects of the tumour may provide a locus for therapeutic intervention in the treatment of solid tumours, if the products of catabolism of host tissues are important in the maintenance of tumour growth.

The principal products released from the degradative activity on adipose tissue and muscle will be free fatty acids (FFA) and amino acids. Tumours have a poor capacity to synthesise their own lipids despite the fact that they are important components of cellular membranes and also play an important role as intracellular mediators such as eicosanoids, diacylglycerol and inositol phospholipids. Nutritional conditions which lead to catabolism of host adipose tissue, such as an acute fast (Sauer & Dauchy, 1987*a*) and acute streptozotocin-induced diabetes (Sauer & Dauchy, 1987*b*), result in a stimulation of tumour growth, suggesting that the products from host fat stores may be limiting for tumour growth *in vivo*. It is most likely that the mitogenic effect results from the release of polyunsaturated fatty acids such as linoleic or arachidonic acids. Phospholipids containing such polyunsaturated fatty acids, esterfied to the sn-2 position of the glycerol moiety, have been shown to stimulate cell growth (Imagawa *et al.*, 1989), possibly by inhibiting a guanosine triphosphatase activating protein, which in turn inactivates the *ras* protein (Tsai *et al.*, 1989). This suggests that these lipids can potentiate *ras* functions.

In addition amino acid requirements are altered in the tumour-bearing state. Host leucine requirements have been shown to be increased in the presence of a rapidly growing murine tumour (Lazo, 1981), and depletion of the nonessential amino acid asparagine by the enzyme asparaginase has led to the treatment of acute lymphocytic leukaemia in man (Holland & Ohnuma, 1979). Some malignant cells have specific requirements for L-cysteine (Uren & Lazarus, 1979), methionine (Tisdale, 1980), tyrosine and phenylalanine (Demetrakopoulos & Brennan, 1982), serine and threonine (Pizer & Reagan, 1972). Removal of one amino acid by the tumour would lead to a depression of host protein synthesis, since normal protein synthesis requires the full complement of amino acids. Disposal of the remaining amino acids has been postulated to explain the abnormal gluconeogenesis seen in cancer patients (Stein, 1978). In view of the extra requirements of the tumour for specific nutrients, it is not surprising that catabolism of host tissues is so common in cancer.

### Model systems of cachexia

In order to understand the underlying mechanisms of cancer cachexia it is important that an appropriate model system is used. Cachexia can appear in patients with tumours which are less than 0.01% of the total body weight (Nathanson & Hall, 1974), and the total tumour mass in the majority of cancer patients rarely exceeds 0.5 Kg (Costa, 1977). Cachexia is also not related to the growth rate or anatomical site of involvement of the tumour. Few of the experimental models which have been used to study the mechanisms of cachexia fulfill the criteria that cachexia should be an early effect of the tumour, and be present with a small tumour burden. Instead cachexia is usually studied in rapidly growing rodent tumours, and appears as a late effect, usually just before death, when the tumour mass has reached a high proportion of the total weight of the animal. Usually these models attribute the cachexia to a drop in food intake and competition of the tumour with the host for essential nutrients (Garattini *et al.*, 1980). The few models which do fulfill the criteria of an early effect and a small tumour mass, observe weight loss without a detectable loss of appetite (Strain *et al.*, 1980; Bibby *et al.*, 1987; Tanaka *et al.*, 1990).

In view of the small tumour mass producing weight loss in such models, it is unlikely that a simple competition between host and tumour is responsible. Indeed, if the tumour-bearing state is compared with pregnancy, the foetus grows at a much faster rate, and is a higher proportion of the total body weight than most tumours. However, in this case the mother actually increases weight. It is also unlikely that weight loss arises solely from an enhanced gluconeogenesis to supply the glucose requirements of the tumour (Gold, 1974), although futile cycles such as the Cori cycle and an increased lipogenesis from glucose probably contribute to the increased metabolic demands in the tumour-bearing state.

### Mechanism of cachexia

We have considered the hypothesis that wasting occurs through direct catabolism of host components by tumour products. There is a considerable amount of data in the literature to support such a hypothesis, particularly with regard to lipid mobilisation. Thus nonviable preparations of Krebs-2 carcinoma cells, when injected into male Swiss mice, were able to produce a decrease in carcass fat of a level comparable with that obtained by viable cell preparations (Costa & Holland, 1966). Using $^{14}CO_2$ production from $^{14}C$ fatty acid labelled adipose tissue as a measure of lipolysis Kitada *et al.* (1980) were able to show that extracts of a thymic lymphoma, as well as serum from mice bearing such a tumour, and culture medium from the cell line growing *in*

Received 11 September 1990; and in revised form 24 October 1990.

*vitro*, were able to mobilise fatty acids from the hosts' adipose tissue. The fact that activity was obtained with the *in vitro* cell line suggested that the lipid mobilising factor was tumour rather than host derived.

Carcass lipid depletion in tumour-bearing nude mice has been shown to be a function of tumour type rather than tumour burden (Hollander *et al.*, 1986), suggesting that only certain tumours are capable of elaborating lipid mobilising factors. In animals bearing a transplantable colon adenocarcinoma (MAC16) weight loss was associated with an elevated plasma lipid mobilising activity (Beck & Tisdale, 1987). Related colon adenocarcinomas which did not produce weight loss had low levels of lipolytic activity, of the order of 10% of that found in the MAC16 tumour. This suggests that the mobilisation of host lipids may be a quantitative phenomonen, with all tumours having some capacity to produce such catabolic factors, and explains why depletion of host lipids is a function of tumour type rather than tumour burden.

### Mediators of cachexia

Considerable effort has been devoted to determining the nature of the circulatory factor present in cachectic animals. Early studies by Kitada *et al.* (1981) suggested that the lipid mobilising factor produced by thymic lymphoma cells was a heat stable protein of molecular weight of about 5 kDa, while later studies reported the active material to be of much higher molecular weight, and to be formed by aggregation of the low molecular weight material (Kitada *et al.*, 1982). A somewhat similar acidic (pI 4.7) lipid mobilising factor of molecular weight 65 to 74 kDa (toxohormone L), has been found in the pleural fluid from patients with malignant lymphoma and the ascites fluids of patients with ovarian carcinoma or hepatoma (Masuno *et al.*, 1981).

Further characterisation of either of these materials has not been reported, although proteolysis of toxohormone L to low molecular weight materials did not destroy lipolytic activity, suggesting some similarity to the low molecular weight material investigated by Kitada *et al.* (1981).

Most recent studies have concentrated on a macrophage product, cachectin, regarded as the mediator of cachexia in animals infected with Trypanosomes (Rouzer & Cerami, 1980) and later shown to be homologous to tumour necrosis factor (TNF) (Beutler *et al.*, 1985*a*). Several studies have demonstrated the ability of TNF to induce weight loss in experimental animals (Oliff *et al.*, 1987). Weight loss produced by TNF is typified by a marked anorexia, and the decrease in food and water intake is directly proportional to the decrease in body weight (Mahony *et al.*, 1988). However, anti-TNF antisera has been shown to delay, but not fully protect against the decrease in food intake in sarcoma-bearing mice (Sherry *et al.*, 1989), suggesting that other factors in addition to TNF may be responsible for the anorexia in tumour-bearing animals. It has been suggested that TNF produces an effect on lipid metabolism through an inhibition of the enzyme lipoprotein lipase (Beutler *et al.*, 1985*b*), and thus depletion of host lipids would be mediated through a different mechanism from the action of the direct catabolic factors mentioned above. Inhibition of lipoprotein lipase by TNF results in hypertriglyceridemia. However, hypertriglyceridemia persists even with tachyphylaxis to the anorectic effects of TNF, suggesting that the two are not inevitably linked (Grunfeld *et al.*, 1989).

Although there is a considerable amount of literature on the biological effects of TNF, the role in cancer cachexia is not clear. Attempts to reverse the cachexia in tumour-bearing animals with anti-TNF antibodies have produced equivocal results (Sherry *et al.*, 1989). Unfortunately, both models used by these workers had a higher tumour burden, and measurements were made at the time of death. In sarcoma-bearing mice anti-TNF antibodies apparently reduced the loss of both carcass and fat mass on the day of death. However, two factors were not taken into consideration. Firstly, animals given anti-TNF antibodies were eating more than control animals when measurements were made, and secondly treatment reduced tumour size, which would also be expected to reduce catabolism of host tissues. In a second study with Lewis lung adenocarcinoma-bearing mice, anti-TNF antibodies diminished the degree of carcass lipid depletion and prevented hypertriglyceridemia. However, as indicated above (Grunfeld *et al.*, 1989) hypertriglyceridemia may not be linked to the anorexia/cachexia syndrome produced by TNF. In neither model did anti-TNF antibodies affect the development of anaemia, hypoalbuminaemia or the increase in serum myeloid P concentration seen with increasing tumour burden. These results suggest that if TNF is involved in the weight loss in some experimental models then other factors must also be present to explain all of the features of cachexia.

Three lines of evidence are required to support the role of a postulated cachectic factor in the development of cachexia.

(1) There should be measurable circulatory levels of the factor and the appearance and level of the material should be correlated with the degree of the cachexia in view of the parabiotic transfer of cachexia in rats (Norton *et al.*, 1985).

(2) Injection of the factor into non weight-losing animals should produce a syndrome similar to the cachectic situation.

(3) Inhibition of the action of the cachectic factor should abolish the cachexia, and may also inhibit tumour growth by deprivation of the tumour of essential nutrients.

### *Correspondence between circulatory levels of cachectic factors and degree of cachexia*

Using tumour cells transfected with the human TNF gene, a correlation has been established between weight loss, anorexia and serum levels of TNF (Oliff *et al.*, 1987). As the authors point out, however, it may be necessary for TNF to be continuously present in the animals circulation for clinically significant changes in metabolic functions to be induced. However, in another murine model of cachexia, the MAC16 adenocarcinoma, where profound cachexia occurs it has been impossible to demonstrate elevated levels of TNF over that found in other tumours which do not induced cachexia, even in the presence of endotoxin (Mahony *et al.*, 1988). Most studies with cancer patients have also reported undetectable serum levels of TNF (Selby *et al.*, 1987), even in patients that had lost eight to 40% of their pre-morbid weight (Socher *et al.*, 1988). Two studies have, however, reported elevated serum levels of TNF, one in children with malignancy (Saarinen *et al.*, 1990), and another in cancer patients with active disease (Balkwell *et al.*, 1987), although in neither study was the level of TNF related to the weight loss and the latter study did not claim to have measured TNF but a 'TNF-like' activity. While the inability to detect TNF in the plasma of cachectic patients may be attributed to the low sensitivity of assays presently available (Beutler & Cerami, 1987), elevated levels of TNF have been detected in other diseases where weight loss occurs, such as the acquired immunodeficiency syndrome (AIDS) (Lahdevirta *et al.*, 1988).

This inability of TNF to be detectable in the serum of cachectic animals/patients has led to the search for other materials which may play a role in the human condition. Using murine adipocytes *in vitro* as a bioassay to detect lipid mobilising activity, we have been able to detect a rise in the plasma level of lipolytic activity in animals bearing the MAC16 adenocarcinoma only when weight loss occurred (Groundwater *et al.*, 1990). The rise in the plasma level of lipolytic activity reached a maximum when the animals had lost 16% of the body weight (Figure 1a), and thereafter declined.

Occassionally animals bearing the MAC16 tumour do not develop weight loss and for these animals there is no increase in plasma lipid mobilising activity (Figure 1b). Using the same assay system the serum lipolytic activity was found to be much higher in cancer patients with weight loss than a group of patients with Alzheimer's disease and comparable

**Figure 1** Changes in serum lipolytic activity (●) with weight loss (□) in **a**, animals bearing the MAC16 tumour which lost weight and in **b**, animals bearing the MAC16 tumour, but without weight loss. Results are expressed as mean ± s.e.m. for four animals per group. Lipolytic activity is expressed as μmoles glycerol released per $10^5$ adipocytes per ml plasma in a 2 h incubation. The average value for non tumour-bearing controls was $0.5 \pm 0.2$ μmoles glycerol $10^5$ adipocytes$^{-1}$ml$^{-1}$.

**Figure 2** Relationship between serum lipolytic activity and weight loss in a group of cancer patients. The lipolytic activity is expressed as in Figure 1. The average value for normal subjects was $0.06 \pm 0.01$ μmoles glycerol $10^5$ adipocytes$^{-1}$ml$^{-1}$.

weight loss (Groundwater *et al.*, 1990). As for animals bearing the MAC16 tumour the serum level of lipolytic activity in cachectic cancer patients was proportional to the degree of weight loss when the total body weight loss did not exceed 20% (Figure 2). Patients who responded to therapy showed a decrease in the plasma levels of lipid mobilising activity, which correlated with the level of response (Beck *et al.*, 1990). The lipid mobilising activity was characterised by the retention by DEAE cellulose, and using this criterion it was observed that similar material was absent from normal serum. This suggests that the lipid mobilising factor is acidic in character, similar to that previously reported (Kitada *et al.*, 1981; Masuno *et al.*, 1981), and is different from the natural lipolytic hormones, which are all basic in character.

### *Effect of cachectic factors on host body weight*

A number of studies have demonstrated the ability of TNF to induce weight loss in experimental animals, although some workers question the ability of TNF alone to yield a sustained cachectic effect (Mullen *et al.*, 1990). In all cases weight loss is accompanied by a marked anorexia, and the depression in food and water intake is probably responsible for the wasting effect. Indeed when the anorexia is counteracted with the synthetic steroid megestrol acetate the ability of TNF to induce weight loss is abolished (Beck & Tisdale, 1990). Megestrol acetate has been shown to produce weight gain in more than 80% of cancer patients administered this agent, with a subjective improvement of appetite occurring in most cases (Aisner *et al.*, 1988). Thus TNF may be responsible for the anorexia commonly encountered in cancer cachexia. However, the cachectic effect of TNF when administered parenterally is usually transient, since tachyphylaxis soon develops. In order to produce a prolonged weight loss escalating doses of TNF are required, which exceed the $LD_{50}$ of the agent (Tracey *et al.*, 1988). Thus experimentally-induced weight loss may be due to the toxic effect of TNF, since a similar condition is observed with the antitumour agent mitozolomide (Mahony & Tisdale, 1988). These results suggest that other factors may be required to explain the weight loss produced by TNF-producing tumours (Oliff *et al.*, 1987).

Treatment of animals with the lipid mobilising factor isolated from the MAC16 tumour also produces a decrease in body weight (Figure 3). This weight loss occurs without an alteration in food intake, and is more pronounced in tumour-bearing animals than in non tumour-bearing animals.

**Figure 3** Effect of a purified extract of culture medium from MAC16 cells containing lipid mobilising activity on the body weight of male NMRI mice (starting weight 25–30 g) bearing the MAC16 tumour, but without weight loss. Animals were administered 100 μl of the purified factor (corresponding to 0.1 μmoles glycerol released $10^5$ adipocytes$^{-1}$ 2 h$^{-1}$) three times daily by i.p. injection (□) and body weights were monitored three times per day. **a**, $P<0.05$; **b**, $P<0.01$; **c**, $P<0.005$ from saline injected controls (◆) by Students *t*-test.

*Relationship between inhibition of postulated cachectic factor and cachexia*

While there has been a large number of studies aimed at determining the role of TNF in cachexia, interestingly very few studies have attempted to reverse the cachexia by antibodies to TNF. In the study of Sherry *et al.* (1989), anti-TNF antibodies did significantly reduce the extent of carcass protein and fat loss in a sarcoma model, as well as the tumour wet and dry weights, but unfortunately measurements were made on the day of death. In the other model investigated, the Lewis lung adenocarcinoma animals did not lose weight, although carcass lipid depletion was partially reversed by anti-TNF antibodies. Until more studies have been performed with other more relevant models of cachexia, the potential use of anti-TNF therapy cannot be evaluated.

*In vitro* studies on the lipid mobilising factor elaborated by the MAC16 tumour showed inhibition by both insulin and 3-hydroxy butyrate (Beck & Tisdale, 1987). Insulin is an important anabolic hormone which has been suggested as a possible supportive measure in the total nutritional management of the cancer patient (Schein *et al.*, 1979). In animals bearing the MAC16 tumour daily insulin administration was shown to reduce host body weight loss, and increase both carcass fat and muscle mass, without an effect on food or water consumption (Beck & Tisdale, 1989*a*). This was only achieved, however, by an increase (about 50%) in the final tumour weight. While in a rat model insulin was shown to produce a significant increase in host body weight without affecting tumour growth (Moley *et al.*, 1985) the known growth stimulatory properties of insulin (Barnes & Sato, 1980) should lead to caution if the use in the nutritional management of cancer patients is being considered.

Medium chain triglycerides (MCT) are usually utilised to induce ketosis *in vivo*, since they are transported directly via the hepatic portal vein to the liver, where they are rapidly oxidised to two carbon units by β-oxidation, and yield high levels of ketone bodies (Cotter *et al.*, 1987). When animals bearing the MAC16 tumour were fed an isocaloric, isonitrogenous diet in which the carbohydrate calories were replaced by lipid in the form of MCT, with up to 80% of the energy derived from MCT, weight loss was reduced by an amount comparable with that obtaied by daily insulin administration, but with a decrease in tumour weight (Tisdale *et al.*, 1987). The effect occurred without an alteration in caloric consumption, and was associated with an increase in both the fat and non-fat carcass mass, and both nitrogen balance and urea excretion were restored to that in non tumour-bearing animals (Beck & Tisdale, 1989*b*). Cancer patients with severe weight loss (mean 32%) also showed an increase in body weight when fed an isocaloric diet in which 70% of the calories were derived from MCT (Fearon *et al.*, 1988). However the effect appeared to occur without an alteration in host nitrogen balance or whole-body protein synthesis or turnover rates. These results suggest that inhibitors of the lipid mobilising factor *in vitro* are also effective inhibitors of cachexia *in vivo*, both in murine models and in cancer patients.

Perhaps the most convincing data has come from recent results which have shown that the most effective reversal of weight loss in mice bearing the MAC16 tumour is achieved when the carbohydrate component of the diet is replaced by lipids derived from fish oil (Tisdale & Dhesi, 1990). In this case weight loss is completely prevented when the fish oil comprised 50% of the total calories and body composition analysis showed that loss of both carcass fat and non-fat mass was completely prevented. Again food intake in weight gaining animals was not changed showing that weight reversal was not due to an effect on energy intake. In this case tumour growth rate was also significantly reduced, although the effect of the fish oil diet on the cachexia exceeded the effect on tumour growth rate, suggesting that the inhibition of the cachexia produced a secondary effect on tumour growth rate.

Fish oil is mainly composed of the essential fatty acids eicospentaenoic (EPA) and docosahexaenoic (DHA) acids. *In vitro* investigations on the individual components of the fish oil showed that EPA was a selective inhibitor of the tumour lipid mobilising factor and tumour-induced proteolysis (Tisdale & Beck, 1991). The inhibitory effect was not seen with DHA, or indeed any other fatty acid of either the (n-3) or (n-6) series. *In vivo* only EPA was effective in inhibiting both weight loss and catabolism of host lipids and proteins, and DHA was totally ineffective. In addition, while *in vitro* studies showed both EPA and DHA to be inhibitors of the growth of the MAC16 tumour, only EPA was effective in inhibiting tumour growth *in vivo* (Tisdale & Beck, 1991). This suggests that the *in vivo* effect of this agent differs from the *in vitro* effect, which is probably due to peroxide formation, and since only EPA has anti-cachectic activity it probably can be attributed to the inhibition of the cachexia.

This suggests that the catabolic effects of the tumour are important in the supply of nutrients essential for tumour growth. The antitumour effect of EPA *in vivo* appears to be reversed by linoleic acid, suggesting that this fatty acid, which is essential for tumour growth, may be supplied by the breakdown of host lipids.

*Mechanisms of action of the tumour lipid mobilising factor at the cellular level*

The catabolic action of most hormones is thought to be mediated through an elevation of intracellular cyclic AMP. In adipocytes this causes activation of protein kinase with the subsequent activation of an inactive form of triglyceride lipase by phosphorylation. Like β-adrenergic stimuli and ACTH the tumour lipid mobilising factor causes an elevation in the intracellular level of cyclic AMP in adipocytes, although unlike the former a prolonged stimulation of cyclic AMP production is observed, in a similar manner to that found with bacterial toxins (Sharp & Hynie, 1971). This suggests that the tumour lipolytic factor may interact directly with guanine nucleotide-binding proteins (G-proteins) involved in signal transduction across plasma membranes.

Stimulation of cyclic AMP levels in adipocytes by β-adrenergic stimuli, ACTH and the tumour lipid mobilising factor are inhibited by EPA, suggesting that the effect is exerted somewhere in the cyclase system. The exact molecular mechanism of action of EPA remains to be identified, but a possible role in the action of G-proteins is shown by the reduced level of ras p21 in mammary tumours from rats fed a fish oil containing diet (Karmali *et al.*, 1989).

### Conclusion

Rapid wasting of body tissues leading to cachexia are characteristic features of a number of diseases, although it is unlikely that there is a similar mediator of cachexia, in the same way that it is simplistic to look for a common cure for cancer. Cachexia is a typical feature of infectious diseases where the invading pathogen may lead to stimulation of the immune system and cytokine production. However, the cachexia of cancer is not normally associated with the presence of infection and while the outward symptoms may look similar, it is unlikely that a single mediator could explain the heterogeneous pattern of changes seen in a wide spectrum of diseases. This raises the possibility that other factors in addition, or instead of the known cytokines, may mediate the changes seen in cancer cachexia. Further structural information of these factors is required for a full understanding of the condition.

The work has been supported by a research grant from the Cancer Research Campaign.

### References

AISNER, J., TCHEKMEDYIAN, S., TAIT, N., PARNES, H. & NOVAK, M. (1988). Studies of high-dose megestrol acetate: potential applications in cachexia. *Seminars in Oncol.*, **15** (Suppl 1), 68.

BARNES, D. & SATO, G. (1980). Methods for growth of cells in serum free medium. *Anal. Biochem.*, **102**, 255.

BALKWELL, F., OSBORNE, K., BURKE, F. & 5 others (1987). Evidence for tumor necrosis factor/cachectin production in cancer. *Lancet*, **ii**, 1229.

BECK, S.A. & TISDALE, M.J. (1987). Production of lipolytic and proteolytic factors by a murine tumor-producing cachexia in the host. *Cancer Res.*, **47**, 5919.

BECK, S.A. & TISDALE, M.J. (1989*a*). Effect of insulin on weight loss and tumour growth in a cachexia model. *Br. J. Cancer*, **59**, 677.

BECK, S.A. & TISDALE, M.J. (1989*b*). Nitrogen excretion in cancer cachexia and its modification by a high fat diet in mice. *Cancer Res.*, **49**, 3800.

BECK, S.A. & TISDALE, M.J. (1990). Effect of megestrol acetate on weight loss induced by tumour necrosis factor alpha and a cachexia-inducing tumour (MAC16) in NMRI mice. *Br. J. Cancer*, **62**, 420.

BECK, S.A., GROUNDWATER, P., BARTON, C. & TISDALE, M.J. (1990). Alteration in serum lipolytic activity in cancer patients with response to therapy. *Br. J. Cancer* (in press).

BEUTLER, B. & CERAMI, A. (1987). Cachetin: more than a tumor necrosis factor. *N. Engl J. Med.*, **316**, 379.

BEUTLER, B., GREENWALD, D., HULMES, J.D. & 5 others (1985*a*). Identity of tumour necrosis factor and the macrophage secreted factor cachectin. *Nature*, **316**, 552.

BEUTLER, B., MAHONEY, J., LETRANG, N., PEKALA, P. & CERAMI, A. (!985*b*). Purificaiton of cachectin, a lipoprotein lipase-suppressing hormone from endotoxin-induced RAW 2647 cells. *J. Exp. Med.*, **161**, 981.

BIBBY, M.C., DOUBLE, J.A., ALI, S.A., FEARON, K.C.H., BRENNAN, R.A. & TISDALE, M.J. (1987). Characteristation of a transplantable adenocarcinoma of the mouse producing cachexia in recipient animals. *J. Natl Cancer Inst.*, **78**, 539.

CHLEBOWSKI, R.T. (1985). Critical evaluation of the role of nutritional support with chemotherapy. *Cancer*, **55**, 268.

COSTA, G. (1977). Cachexia, the metabolic component of neoplastic diseases. *Cancer Res.*, **37**, 2327.

COSTA, G. & HOLLAND, J.F. (1966). Effects of Krebs-2 carcinoma on the lipid metabolism of male Swiss mice. *Cancer Res.*, **22**, 1681.

COTTER, R., TAYLOR, C.A., JOHNSON, R. & ROWE, W.B. (1987). A metabolic comparison of pure long-chain triglyceride emulsions (LCT) and various medium chain triglycerides (MCT) - LCT combination emlusions in dogs. *Am. J. Clin. Nutr.*, **45**, 927.

DEMETRAKOPOULOS, G.E.V. & BRENNAN, M.F. (1982). Tumoricidal potential of nutritional manipulations. *Cancer Res.*, **42** (Suppl.), 756s.

DE WYS, W.D. (1985). Management of cancer cachexia. *Seminars in Oncol.*, **12**, 452.

FEARON, K.C.H., BORLAND, W., PRESTON, T., TISDALE, M.J., SHENKIN, A. & CALMAN, K.C. (1988). Cancer cachexia: influence of systemic ketosis on substrate levels and nitrogen metabolism. *Am. J. Clin. Nutr.*, **47**, 42.

GARATTINI, S., BIZZI, A., DONELLI, M.G., GUAITANI, A., SAMANIN, R. & SPREAFICO, F. (1980). Anorexia and cancer in animals and man. *Cancer Treat. Rev.*, **7**, 115.

GOLD, J. (1974). Cancer cachexia and gluconeogenesis. *Ann. NY Acad. Sci.*, **230**, 103.

GROUNDWATER, P., BECK, S.A., BARTON, C., ADAMSON, C., FERRIER, I.N. & TISDALE, M.J. (1990). Alterations of serum and urinary lipolytic activity with weight loss in cachectic cancer patients. *Br. J. Cancer*, **62**, 816.

GRUNFELD, C., WILKING, H., NEESE, R. & 5 others (1989). Persistence of the hypertriglyceridemic effect of tumor necrosis factor despite development of tachyphylaxis to the anorectic/cachectic effects in rats. *Cancer Res.*, **49**, 2554.

HOLLAND, J.F. & OHNUMA, T. (1979). Lessons from the study of induced alterations in amino acids in patients with cancer. *Cancer Treat. Rep.*, **63**, 1013.

HOLLANDER, M.D., EBERT, E.C., ROBERTS, A.I. & DEVEREUX, D. (1986). Effect of tumor type and burden on carcass lipid depletion in mice. *Surgery*, **100**, 292.

IMAGAWA, W., BANDYOPADHYAY, G.K., WALLACE, D. & NANDI, S. (1989). Phospholipids containing fatty acyl groups are mitogenic for normal mouse mammary epithelial cells in serum-free primary cell culture. *Proc. Natl Acad. Sci. USA*, **86**, 4122.

KARMALI, R.A., CHAO, C.-C., BASU, A. & MODAK, M. (1989). Effect of n-3 and n-6 fatty acids on mammary H-ras expression and $PGE_2$ levels in DMBA-treated rats. *Anticancer Res.*, **9**, 1169.

KITADA, S., HAYS, E.F. & MEAD, J.F. (1980). A lipid mobilizing factor in serum of tumor-bearing mice. *Lipids*, **15**, 168.

KITADA, S., HAYS, E.F. & MEAD, J.F. (1981). Characterization of a lipid mobilizing factor from tumors. *Prog. Lipid Res.*, **28**, 823.

KITADA, S., HAYS, E.F., MEAD, J.F. & ZABIN, I. (1982). Lipolysis induction in adipocytes by a protein from tumor cells. *J. Cell Biochem.*, **20**, 409.

LAHDEVIRTA, J., MAURY, C.P.J., TEPPO, A.-M. & REPO. H. (1988). Elevated levels of circulating cachectin/tumor necrosis factor in patients with acquired immunodeficiency syndrome. *Am. J. Med.*, **85**, 289.

LAZO, P.A. (1981). Tumour induction of host leucine starvation. *FEBS Lett.*, **135**, 229.

MAHONY, S.M. & TISDALE, M.J. (1988). Induction of weight loss and metabolic alterations by human recombinant tumour necrosis factor. *Br. J. Cancer*, **58**, 345.

MAHONY, S.M., BECK, S.A. & TISDALE, M.J. (1988). Comparison of weight loss induced by recombinant tumour necrosis factor with that produced by a cachexia-inducing tumour. *Br. J. Cancer*, **57**, 385.

MASUNO, H., YAMASAKI, N. & OKUDA, H. (1981). Purification and characterization of lipolytic factor (toxohormone-L) from cell-free fluid of ascites sarcoma 180. *Cancer Res.*, **42**, 284.

MOLEY, J.F., MORRISON, S.D., GORSCHBOTH, C.M. & NORTON, J.A. (1988). Body composition changes in rats with experimental cancer cachexia: improvement with exogenous insulin. *Cancer Res.*, **48**, 2784.

MULLEN, B.J., HARRIS, R.B.S., PATTON, J.S. & MARTIN, R.J. (1990). Recombinant tumor necrosis factor-α chronically administered in rats: lack of cachectic effect. *Proc. Soc. Exp. Biol. Med.*, **193**, 318.

NATHANSON, L. & HALL, T.C. (1974). A spectrum of tumors that produce paraneoplastic syndromes. *Ann. NY Acad. Sci.*, **230**, 367.

NORTON, J.A., MOLEY, J.F., GREEN, M.V., CARSON, R.E. & MORRISON, S.D. (1985). Parabiotic transfer of cancer anorexia/cachexia in male rats. *Cancer Res.*, **45**, 5547.

OLIFF, A., DEFO-JONES, D., BOYER, M. & 5 others (1987). Tumors secreting human TNF/cachectin induce cachexia in mice. *Cell*, **50**, 555.

PIZER, L.I. & REGAN, J.D. (1972). Basis for the serine requirement in leukemic and normal human leukocytes. Reduced levels of the enzymes in the phosphorylated pathway. *J. Natl Cancer Inst.*, **48**, 1897.

ROUZER, C.A. & CERAMI, A. (1980). Hypertriglyceridemia associated with Trypanosoma brucei brucei infection in rabbits: roll of defective triglyceride removal. *Mol. Biochem. Parasitol.*, **2**, 31.

SAARINEN, U.M., KOSKELO, E.K., TEPPO, A.M. & SIIMES, M.A. (1990). Tumor necrosis factor in chidren with malignancies. *Cancer Res.*, **50**, 592.

SAUER, L.A. & DAUCHY, R.T. (1987*a*). Blood nutrient concentrations and tumor growth *in vivo* in rats: relationships during the onset of an acute fast. *Cancer Res.*, **47**, 1065.

SAUER, L.A. & DAUCHY, R.T. (1987*b*). Stimulation of tumor growth in adult rats *in vivo* during acute streptozotocin-induced diabetes. *Cancer Res.*, **47**, 1756.

SCHEIN, P.S., KISNER, D., HALLER, D., BELCHER, M. & HAMOSH, M. (1979). Cachexia of malignancy. Potential role of insulin in nutritional management. *Cancer*, **43**, 2070.

SELBY, P., HOBBS, S., VINER, C. & 7 others (1987). Tumour necrosis factor in man: clinical and biological observations. *Br. J. Cancer*, **56**, 803.

SHARP, G.W.G. & HYNIE, S. (1971). Stimulation of intestinal adenyl cyclase by cholera toxin. *Nature*, **229**, 266.

SHERRY, B.A., GELIN, J., FONG, Y. & 6 others (1989). Anticachectin/tumor necrosis factor-α antibodies attentuate development of cachexia in tumor models. *FASEB J.*, **3**, 1956.

SOCHER, S.H., MARTINEZ, D., CRAIG, J.B., KUHN, J.G. & OLIFF, A. (1988). Tumor necrosis factor not detectable in patients with clinical cancer cachexia. *J. Natl Cancer Inst.*, **80**, 595.

STEIN, T.P. (1978). Cachexia, gluconeogenesis and progressive weight loss in cancer patients. *J. Theoret. Biol.*, **73**, 51.

STRAIN, A.J., EASTY, G.C. & NEVILLE, A.M. (1980). An experimental model of cachexia induced by a xenografted human tumor. *Cancer Res.*, **64**, 217.

STUDLEY, H.O. (1936). Percentage of weight loss, a basic indicator of surgical risk. *J. Amer. Med. Assoc.*, **106**, 458.

TANAKA, Y., EDA, H., TANAKA, T. & 6 others (1990). Experimental cancer cachexia induced by transplantable colon 26 adenocarcinoma in mice. *Cancer Res.*, **50**, 2290.

TISDALE, M.J. (1980). Effect of methionine replacement by homocysteine on the growth of cells. *Cell Biol. Int. Rep.*, **4**, 563.

TISDALE, M.J., BRENNAN, R.A. & FEARON, K.C. (1987). Reduction of weight loss and tumour size in a cachexia model by a high fat diet. *Br. J. Cancer*, **56**, 39.

TISDALE, M.J. & BECK, S.A. (1991). Inhibition of tumour-induced lipolysis *in vitro* and cachexia and tumour growth *in vivo* by eicosapentaenoic acid. *Biochem. Pharmacol.*, **41**, 103.

TISDALE, M.J. & DHESI, J.K. (1990). Inhibition of weight loss by ω-3 fatty acids in an experimental cachexia model. *Cancer Res.*, **50**, 5022.

TRACEY, K.J., WEI, H.E., MANOGUE, K.R. & 8 others (1988). Cachectin/tumor necrosis factor induces cachexia, anemia and inflammation. *J. Exp. Med.*, **167**, 1211.

TSAI, M.-H., YU, C.-L., WEI, F.-S. & STACEY, D.W. (1989). The effect of GTPase activating protein upon Ras is inhibited by mitogenically responsive lipids. *Science*, **243**, 522.

UREN, J.R. & LAZARUS, H. (1979). L-Cyst(e)ine requirements of malignant cells and progress toward depletion therapy. *Cancer Treat. Rep.*, **63**, 1073.

VAN EYS, J. (1982). Effect of nutritional status on response to therapy. *Cancer Res.*, **42** (Suppl), 747.

WARREN, S. (1932). The immediate causes of death in cancer. *Am. J. Med. Sci.*, **184**, 610.